# Tiered Functional Screening Identifies an Autochthonous Vaginal *Lactiplantibacillus plantarum* Strain with Probiotic Potential

**DOI:** 10.3390/microorganisms14071526

**Published:** 2026-07-13

**Authors:** Viktoria Nazarova, Nazira Kamzayeva, Samat Kozhakhmetov, Almagul Kushugulova, Milan Terzic, Gauri Bapayeva, Berik Primbetov, Balkenzhe Imankulova, Gulzhanat Aimagambetova, Yevgeniy Kim, Kuralay Kongrtay, Nazira Kadroldinova, Makhabbat Galym, Sanimkul Makhambetova, Kadisha Nurgaliyeva, Zhanar Abdiyeva, Zhanar Zhumakanova, Saule Akhmetova, Zhanerke Amirkhanova, Balnur Smagulova, Aidana Tastanova, Talshyn Ukybassova

**Affiliations:** 1 Laboratory of Microbiome, Center for Life Sciences, National Laboratory Astana, Nazarbayev University, Astana 010000, Kazakhstan; semeykz@gmail.com (V.N.);; 2 Clinical Academic Department of Women’s Health, Corporate Fund “University Medical Center”, Astana 010000, Kazakhstan; milan.terzic@nu.edu.kz (M.T.); kkongrtay@nu.edu.kz (K.K.); mahabbat_g@mail.ru (M.G.); sanimkul.makhambetova@umc.org.kz (S.M.);; 3 Department of Surgery, School of Medicine, Nazarbayev University, Astana 010000, Kazakhstan; 4 Department of Biomedicine, Karaganda Medical University, Karaganda 100000, Kazakhstan; 5 Department of Obstetrics and Gynecology No. 1, Astana Medical University, Astana 010000, Kazakhstan; 202514637@amu.kz; 6 School of Sciences and Humanities, Nazarbayev University, Astana 010000, Kazakhstan; aidana.tastanova@nu.edu.kz

**Keywords:** cervicovaginal microbiome, *Lactiplantibacillus plantarum*, autochthonous probiotic, biofilm formation, vaginal lactobacilli, HPV, Central Asia, strain-level functional screening

## Abstract

Persistent high-risk human papillomavirus (HPV) infection drives cervical cancer, a leading cause of cancer-related mortality among women in low- and middle-income countries; its clinical course is shaped by the cervicovaginal microbiome, in which *Lactobacillus*-dominated communities are associated with enhanced viral clearance. Despite this, vaginal probiotic interventions often demonstrate limited colonization efficiency, and autochthonous strain libraries from Central Asia remain absent. We applied a tiered functional screening workflow to a collection of 235 vaginal lactic acid bacterial isolates recovered from 400 women undergoing routine gynecological examination in Astana, Kazakhstan. The workflow sequentially filtered isolates on (i) antimicrobial activity against seven urogenital indicator pathogens using the deferred antagonism assay, (ii) surface adhesion by the Brilis erythrocyte assay, and (iii) biofilm-forming capacity by crystal violet retention and laser-capture-microdissection (LCM) microscopy. Species-level identification of the selected candidate was performed by whole-genome shotgun sequencing followed by Kraken2 taxonomic classification. From 235 isolates, three rounds of phenotypic filtering identified four broad-spectrum antimicrobial candidates (127-3, 127-4, 107-2, 107-4) with non-overlapping inhibitory profiles against seven urogenital indicator strains. Adhesion phenotyping segregated candidates into low- and moderate-adhesion groups, with none reaching the high-adhesion threshold. Among all four candidates, only strain 127-4 produced a reproducible biofilm-associated signal (crystal violet retention OD490 = 0.09 ± 0.07 at 24 h; 0.08 ± 0.03 at 48 h), consistent with early surface attachment under static conditions. Whole-genome shotgun sequencing assigned 97.81% of classified reads to *Lactiplantibacillus plantarum*, supporting preliminary identification of the selected isolate as *L. plantarum* strain 127-4. Composite ranking confirmed 127-4 as the only isolate combining broad antimicrobial activity (5/7 indicators), moderate adhesion (specific adhesion index, SPA = 2.95), and a detectable biofilm-associated phenotype. We report the first systematic functional screening of autochthonous cervicovaginal lactic acid bacteria from a Central Asian population and identify *L. plantarum* 127-4 as a probiotic candidate with an integrated trait profile rarely identified through single-criterion screening approaches. Beyond candidate identification, this work establishes a transferable workflow for assembling functionally annotated vaginal *Lactobacillus* collections from underrepresented populations, providing a foundation for future population-specific probiotic interventions targeting cervicovaginal health.

## 1. Introduction

Probiotic interventions for vaginal health have, paradoxically, been built largely on strains isolated from dairy fermentations rather than from the host niche they aim to restore [1]. This mismatch between source and target ecosystems may help explain why, despite three decades of clinical investigation, the colonization efficiency and durable effect size of vaginal probiotics remain modest and population-dependent [2]. Closing this gap requires systematic functional characterization of autochthonous vaginal lactic acid bacteria—work that has been performed almost exclusively in European, North American, and East Asian cohorts, leaving the populations bearing the heaviest cervical cancer burden essentially unrepresented in current strain libraries.

The clinical stakes are substantial. Cervical cancer remains the fourth most common malignancy and the fourth leading cause of cancer death in women, with 660,000 new cases and 350,000 deaths in 2022, more than 90% concentrated in low- and middle-income countries [3]. Persistent infection with high-risk human papillomavirus (HPV) is the necessary etiological step, but only a minority of HPV infections persist—an outcome shaped, in addition to viral genotype and host immunity, by the cervicovaginal microbiome. Communities dominated by *Lactobacillus crispatus* (community state type [CST] I) are associated with HPV clearance rates substantially higher than those observed in CST IV communities depleted of lactobacilli and enriched for *Gardnerella*, *Atopobium*, *Prevotella*, and *Sneathia* [4,5]. Mechanistically, lactobacilli enforce mucosal homeostasis through acidification of the vaginal lumen via D- and L-lactic acid, suppression of pro-inflammatory IL-1β/IL-17 signaling that otherwise facilitates HPV-driven epithelial transformation, and competitive exclusion of pathobionts at the mucin–epithelium interface [6,7]. These mechanisms motivate—but do not yet operationalize—the rational selection of vaginal probiotic strains.

Three knowledge gaps currently prevent the translation of these insights into deployable interventions. First, the protective phenotype is not a property of *Lactobacillus* as a species but of specific strains: biofilm-forming capacity, lactic acid production rate, bacteriocin output, and adhesion strength can vary by an order of magnitude or more among isolates within the same species, including *L. crispatus*, *L. gasseri*, and *Lactiplantibacillus plantarum* [8,9]. Species-level taxonomy—the resolution at which most current probiotic candidates are selected—is therefore an inadequate predictor of clinical performance. Second, biofilm formation, increasingly recognized as the principal determinant of mucosal persistence and the key trait differentiating colonizing from transient strains [10,11], is rarely incorporated into selection workflows alongside antimicrobial activity and adhesion. Third, autochthonous vaginal *Lactobacillus* collections have been built almost exclusively from European, North American, and East Asian populations, despite documented inter-population variation in CST distribution, *Lactobacillus* species composition, and HPV epidemiology. To our knowledge, no functionally characterized vaginal *Lactobacillus* strain bank from Central Asia—a region with cervical cancer incidence reaching 18.7 per 100,000 in Kazakhstan, more than twice the Western European average [12]—has been reported. This absence is not a parochial concern: population-specific strains are increasingly understood to be a prerequisite for effective probiotic colonization, given the co-evolved nature of microbiome–host interactions [13].

These gaps are tractable—but only with a screening framework designed around them. Most published vaginal probiotic candidates have been selected on a single dominant criterion (typically antimicrobial activity), with adhesion and biofilm capacity assessed post hoc, if at all. Such single-axis selection systematically discards strains whose value lies in trait combinations rather than in a single peak phenotype. A tiered, sequential screen—filtering first on antimicrobial activity against urogenital pathogens, then on adhesion, then on biofilm-forming capacity—provides a tractable alternative that surfaces strains optimized at the level of the integrated colonization phenotype. Antimicrobial activity, epithelial adhesion, and biofilm-associated persistence are widely regarded as core functional determinants of vaginal probiotic performance and mucosal colonization capacity. No universally accepted ranking system currently exists for prioritizing vaginal probiotic candidates. Consequently, recent studies have increasingly adopted multi-trait screening approaches that integrate complementary functional characteristics, including pathogen inhibition, adhesion capacity, and persistence-associated phenotypes, to support rational strain selection. In the present study, these principles were adapted to construct an exploratory composite profile for comparative prioritization of shortlisted isolates.

Because these traits frequently vary independently even among isolates of the same species, multi-trait screening frameworks have increasingly been advocated for rational vaginal probiotic prioritization [2,8,9,10].

Here, we apply this framework to a cohort of 400 women undergoing routine gynecological examination in Astana, Kazakhstan—to our knowledge, the first systematic functional screen of autochthonous cervicovaginal lactic acid bacteria from a Central Asian population. From 235 isolates, three rounds of phenotypic filtering identified *Lactiplantibacillus plantarum* strain 127-4 as combining pronounced antimicrobial activity against seven urogenital indicator pathogens, moderate adhesion to host-relevant cellular substrates, and detectable biofilm formation—an integrated trait profile rarely captured by single-criterion screens. Beyond strain identification, the resulting framework establishes a transferable workflow for assembling functionally annotated vaginal *Lactobacillus* collections from underrepresented populations, providing a foundation for the population-specific probiotic interventions that next-generation cervicovaginal health strategies will require.

## 2. Materials and Methods

### 2.1. Study Population and Ethics

Vaginal swabs were collected from 400 adult women (mean age 34.5 ± 6.4 years; range 19.9–46.0) attending routine gynecological examination at the Clinical Academic Department of Women’s Health, Corporate Fund “University Medical Center” (UMC), Astana, Kazakhstan, between January and March 2025. The study was conducted in accordance with the Declaration of Helsinki and approved by the Local Bioethics Committee of UMC (Protocol No. 5; 23 May 2024). Written informed consent was obtained from all participants prior to sample collection. Age and HPV status data were available from clinical records; HPV-stratified analyses are reported in a companion population-level study.

### 2.2. Isolation and Preliminary Identification of Vaginal Isolates

Vaginal samples were plated on de Man, Rogosa and Sharpe (MRS) agar (Merck, Darmstadt, Germany) and incubated at 37 °C for 48 h under anaerobic conditions (Anoxomat system, Mart Microbiology, Lichtenvoorde, The Netherlands). Following incubation, colonies were selected based on morphological characteristics consistent with the genus *Lactobacillus*. Preliminary identification of the isolates was performed by assessing catalase activity, Gram staining, and microscopic examination of cellular morphology. A total of 235 Gram-positive, catalase-negative isolates putatively belonging to the genus *Lactobacillus* were obtained from the 400 clinical samples (recovery rate 58.8%) and advanced to the stepwise screening workflow.

### 2.3. Antimicrobial Activity Assay

Antimicrobial activity was assessed by the deferred antagonism double-agar overlay assay with minor modifications [14,15]. Producer isolates were inoculated by stabbing with a bacteriological loop onto MRS agar plates and incubated at 37 °C for 48 h under anaerobic conditions. Producer cultures were then exposed to chloroform vapor for 5 min to inactivate viable cells before overlay. Plate surfaces were overlaid with 5 mL of molten nutrient medium cooled to 45 °C and previously mixed with one of the seven indicator microorganisms: *Streptococcus agalactiae* ATCC 13813, *Proteus mirabilis* ATCC 25933, *Candida glabrata* ATCC 15126, *Escherichia coli* ATCC 25922, *Staphylococcus aureus* ATCC 29213, *Enterococcus faecalis* ATCC 19433, and *Candida albicans* ATCC 10231. Plates were incubated at 37 °C for 24 h under aerobic or anaerobic conditions according to the indicator organism’s requirements.

Inhibition zones were measured as the total diameter (mm) of the visible growth inhibition halo surrounding the producer colony. Two perpendicular measurements were performed and the mean value was used for quantitative assessment. Antimicrobial activity was classified as: ≤1.0 mm—no activity; 1.1–4.9 mm—low activity; 5.0–8.9 mm—moderate activity; ≥9.0 mm—high activity. All experiments were performed in three independent replicates. We acknowledge that the deferred antagonism format does not differentiate the relative contributions of organic acid production, hydrogen peroxide, and bacteriocin-like inhibitory substances to the observed inhibition; resolution of this question through pH-controlled and neutralized cell-free supernatant assays is planned as part of the strain’s mechanistic characterization.

### 2.4. Adhesion Assay

Adhesive activity of selected isolates was evaluated using the Brilis erythrocyte adhesion assay [16] as a comparative screening proxy for surface adhesion. Washed human erythrocytes obtained from the Republican Scientific and Production Center of Transfusiology (Astana, Kazakhstan) were used, with samples processed in anonymized form. A drop of physiological saline was placed on a glass slide, into which one inoculating loop of erythrocyte suspension and one loop of the tested bacterial culture freshly collected from the growth medium were mixed. Preparations were incubated in a humid chamber at 37 °C for 30 min, dried at the same temperature, heat-fixed, Gram-stained, and examined by light microscopy.

Each experiment was performed in three independent replicates. The mean number of bacterial cells adhering to a single erythrocyte (specific adhesion index, SPA) was calculated by analyzing at least 25 erythrocytes, with no more than five erythrocytes per microscopic field. Adhesion was classified as: SPA 0–1.0—no adhesion; 1.01–2.0—low adhesion; 2.01–4.0—moderate adhesion; >4.0—high adhesion.

### 2.5. Biofilm Formation Assay

Biofilm formation by the four shortlisted isolates was assessed using the O’Toole microtiter plate assay with minor modifications [17]. Sterile 96-well flat-bottom polystyrene microplates were used. Each well received 150 µL of MRS broth and 50 µL of bacterial suspension standardized to OD_490_ = 0.1. Each strain was tested in four parallel wells, with control wells containing MRS medium only. Plates were incubated at 37 °C under anaerobic conditions for 24 and 48 h.

After incubation, the planktonic phase was removed and wells were gently washed three times with phosphate-buffered saline (pH 7.2). Plates were incubated at 60 °C for 60 min to fix the attached biofilms. Biofilms were stained with 2% crystal violet (150 µL per well) for 15 min at room temperature, and excess dye was removed. The bound dye was eluted with 95% ethanol (150 µL per well) for 30 min, and absorbance was measured at 490 nm using the available microplate reader. All measurements were performed in three independent experimental series. Reference biofilm-forming and non-biofilm-forming control strains were not included in the present comparative assay; this limitation is addressed in the Discussion. Although wavelengths of 570 or 595 nm are more commonly used in published crystal violet assays, OD_490_ was applied consistently to all samples in the present study, allowing valid within-study comparisons but limiting cross-study quantitative comparability [18].

### 2.6. Biofilm-Associated Imaging by Laser-Capture Microdissection

Biofilm-associated structures formed by the priority isolate *L. plantarum* 127-4 on glass surfaces were visualized using a modified protocol [18]. Sterile glass slides were placed in 100 mm Petri dishes and covered with 1 mL of bacterial suspension standardized to 1.0 McFarland. After 4 h at 37 °C for initial cell attachment, 5 mL of MRS broth was added and cultivation continued under anaerobic conditions for 24 and 48 h. Slides were rinsed with phosphate buffer to remove non-adherent cells, fixed with 96% ethanol, stained with 2% crystal violet, and mounted using Vitrogel (Biovitrum, St. Petersburg, Russia). Surface-associated structures were visualized using the ArcturusXT™ Laser Capture Microdissection system (Thermo Fisher Scientific, Waltham, MA, USA). The approach provides qualitative visualization of surface-associated bacterial aggregates; mature biofilm architecture cannot be inferred from these images and requires confocal laser scanning microscopy with matrix-component staining (e.g., concanavalin A; SYTO9/propidium iodide).

### 2.7. Whole-Genome Shotgun Sequencing and Taxonomic Classification

Reliable species-level identification is a prerequisite for any candidate advanced toward probiotic application, in line with the FAO/WHO and ISAPP minimum standards for probiotic strain characterization [19,20,21]. Prior to molecular analysis, isolate 127-4 was purified by repeated subculturing on MRS agar until morphologically homogeneous colonies were obtained. Genomic DNA was extracted from a purified colony using the ZymoBIOMICS™ DNA Miniprep Kit (Zymo Research Corporation, Irvine, CA, USA) according to the manufacturer’s instructions, with bead-beating lysis in ZR BashingBead™ tubes (Zymo Research Corporation, Irvine, CA, USA) followed by column-based DNA purification. DNA was eluted in DNase/RNase-free water and further purified using Zymo-Spin™ III-HRC filters (Zymo Research Corporation, Irvine, CA, USA).

Whole-genome shotgun sequencing was performed on the DNBSEQ platform using paired-end 150 bp reads (PE150). For strain 127-4, a total of 24,141,435 quality-filtered reads were obtained, corresponding to 7,242,430,500 clean bases. Raw reads were quality-filtered, and sequencing reads were taxonomically classified using Kraken2 (v2.1.3) [22] against the standard bacterial reference database. The sequence data that support the findings of this study have been deposited in the NCBI Sequence Read Archive under BioProject accession number PRJNA1470428.

### 2.8. Composite Functional Ranking

To document the candidate-selection logic of the tiered screen, we generated a composite functional profile across the four shortlisted candidates incorporating four parameters relevant to vaginal probiotic application: (i) breadth of antimicrobial activity (number of indicators inhibited at ≥5 mm halo diameter); (ii) maximum inhibition halo against any single indicator; (iii) SPA value as a proxy for surface adhesion; (iv) biofilm-associated OD_490_ at 48 h. Values are reported in Supplementary Table S2. The composite framework was intended as an exploratory multi-parameter prioritization tool rather than a validated clinical scoring system.

### 2.9. Statistical Analysis

The present study was designed as an exploratory screening workflow aimed primarily at candidate prioritization rather than formal hypothesis testing among strains. Data are therefore reported as descriptive statistics (mean ± standard deviation) of three independent replicates. Given the limited biological replication and the predominance of near-baseline or negative blank-corrected OD_490_ values among non-biofilm-forming candidates, interpretation focused primarily on descriptive comparative trends and reproducibility across independent experiments. Standard deviations were calculated using GraphPad Prism v9 (GraphPad Software, San Diego, CA, USA).

## 3. Results

### 3.1. Cohort and Recovery of Presumptive Vaginal Lactobacilli

Vaginal swabs were obtained from 400 adult women (mean age 34.5 ± 6.4 years; range 19.9–46.0) attending routine gynecological examination at the University Medical Center, Astana, Kazakhstan. Direct plating on MRS agar followed by anaerobic incubation at 37 °C for 48 h yielded 235 Gram-positive, catalase-negative isolates with rod-shaped morphology consistent with the genus *Lactobacillus*, corresponding to a recovery rate of 58.8% per swab. All isolates produced small (1–3 mm), non-pigmented, circular colonies with smooth surfaces and regular margins; representative colonial and microscopic morphology is shown in Supplementary Figure S1. The full set of 235 presumptive isolates was advanced to the tiered functional screening workflow described below, which prioritized antagonistic activity, adhesion capacity, and biofilm-associated phenotype as sequential selection criteria for probiotic candidate identification.

### 3.2. Antimicrobial Activity Against Urogenital Indicator Strains

**Figure 1:**
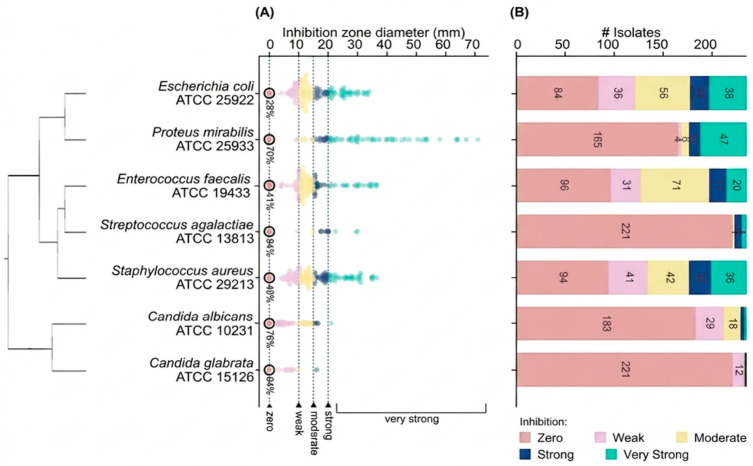
Antimicrobial activity profiles of vaginal *Lactobacillus* isolates against indicator strains. (**A**) Distribution of inhibition zone diameters (mm) produced by the tested isolates against seven indicator strains (*Escherichia coli* ATCC 25922, *Proteus mirabilis* ATCC 25933, *Enterococcus faecalis* ATCC 19433, *Streptococcus agalactiae* ATCC 13813, *Staphylococcus aureus* ATCC 29213, *Candida albicans* ATCC 10231, and *Candida glabrata* ATCC 15126). Dashed vertical lines indicate descriptive activity bins (zero, weak, moderate, strong, and very strong) used to visualize inhibition-zone distributions across the isolate collection. These categories were used for descriptive visualization purposes only and were not applied as operational screening thresholds during isolate prioritization. (**B**) Number of isolates exhibiting different levels of antimicrobial activity against each indicator strain.

Antimicrobial activity of all 235 isolates was evaluated against seven urogenital indicator strains using the deferred antagonism double-agar overlay assay in three independent replicates. Inhibitory activity was strongly stratified by indicator (Figure 1A,B).

The largest fraction of active isolates was observed against *P. mirabilis* (64% of the panel), followed by *E. coli* and *S. aureus* (30% each), *E. faecalis* (26%), *C. albicans* (22%), *S. agalactiae* (16%), and *C. glabrata* (5%). The marked stratification between Gram-negative and Gram-positive/yeast indicators is consistent with previous reports for vaginal lactobacilli [1,11] and likely reflects the dominant contribution of organic acid–mediated inhibition in the deferred antagonism format, alongside potentially target-selective antimicrobial metabolites whose contribution warrants further investigation (see Section 4).

For each Gram-negative indicator, the distribution of inhibition zone diameters was bimodal rather than continuous, with a numerically small subpopulation of high-activity producers (≥9 mm halos) standing out from a majority of weak or moderate producers. Based on this primary screening stage, 37 isolates were retained on the basis of either high single-target activity (≥9 mm) or activity against three or more indicators. The ≥9 mm criterion was used exclusively as a retention threshold during candidate selection, whereas the activity categories displayed in Figure 1A were included solely for descriptive visualization of inhibition-zone distributions and were not applied during isolate prioritization.

**Figure 2:**
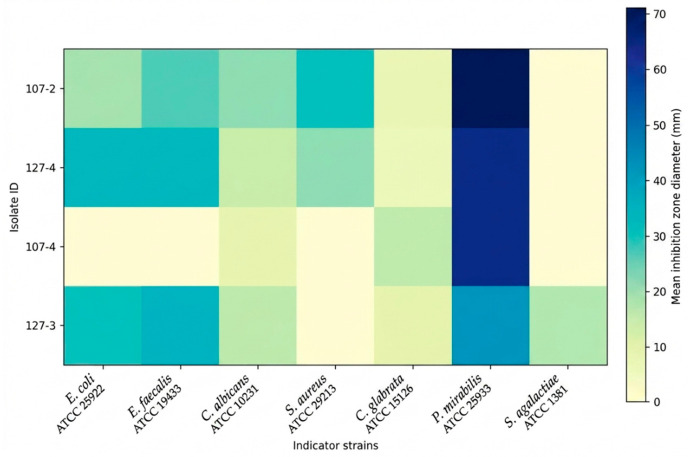
Heatmap of mean inhibition zone diameters (mm) for four selected isolates identified through stepwise screening of 235 vaginal cultures. Color intensity ranges from light yellow (lower values) to dark blue (higher values). Values represent three independent experiments.

From this subset, four isolates—127-3, 127-4, 107-2, and 107-4— displayed the broadest antimicrobial spectrum (≥4 of 7 indicators) combined with the largest mean halo diameters and were therefore prioritized for comparative functional characterization. Comparative antimicrobial activity profiles of the four shortlisted strains are visualized in Figure 2, while the complete activity profiles of all 37 retained isolates are provided in Supplementary Table S1.

The four shortlisted candidates exhibited distinct antimicrobial profiles. Strain 127-4 produced the largest inhibition halos against *E. coli* (33.7 ± 0.6 mm) and *E. faecalis* (33.5 ± 0.5 mm); strain 127-3 produced the largest halo against *E. faecalis* (35.8 ± 2.0 mm) and was the only candidate active against *S. agalactiae* (17.5 ± 0.5 mm); strain 107-2 demonstrated the strongest anti-*S. aureus* antimicrobial activity (30.5 ± 0.5 mm); and strain 107-4 produced the largest anti-yeast halo against *C. glabrata* (16.0 ± 0.5 mm). All four candidates produced large inhibition halos (>43 mm) against *P. mirabilis*. The complementary nature of these antimicrobial profiles indicated that no single candidate dominated across all indicator strains and that selection of a probiotic-relevant isolate required integration with adhesion- and biofilm-associated phenotypes, consistent with the multi-trait selection framework increasingly advocated for vaginal probiotic development [2,20].

### 3.3. Surface Adhesion Phenotype Stratifies Candidates into Two Functional Groups

**Figure 3:**
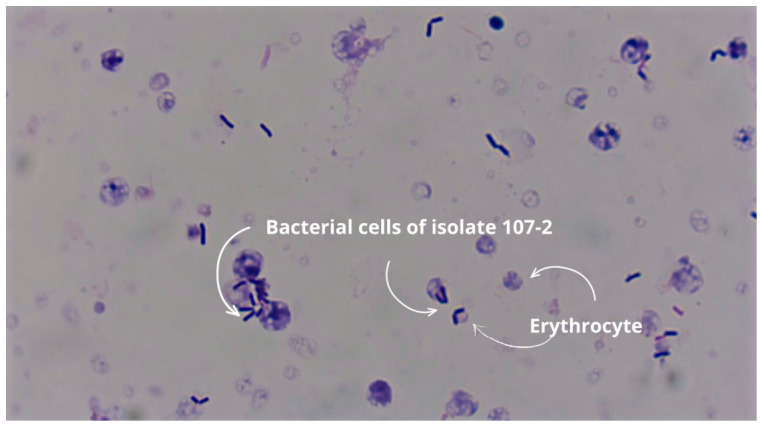
Adhesion of vaginal *Lactobacillus* isolate 107-2 to erythrocytes assessed by the Brilis assay. Representative image showing adherent bacterial cells of isolate 107-2 on erythrocytes following incubation (light microscopy, ×1000).

The four shortlisted candidates were next evaluated for surface adhesion using the Brilis erythrocyte adhesion assay as a screening proxy (Figure 3) [16].

The mean SPA across the four candidates ranged from 1.81 ± 0.24 to 3.39 ± 0.62, with no candidate reaching the high-adhesion threshold (SPA > 4.0). The candidates segregated into two distinct adhesion phenotypes: low adhesion (107-2: 1.81 ± 0.24; 127-3: 1.86 ± 0.46) and moderate adhesion (127-4: 2.95 ± 0.53; 107-4: 3.39 ± 0.62). Based on these values, strains 107-2 and 127-3—both isolates demonstrating strong antimicrobial activity in the previous screening tier—were considered less favorable as mucosal-persistence candidates, whereas 127-4 and 107-4 demonstrated the strongest adhesion-associated phenotypes prior to biofilm evaluation.

We note that the erythrocyte adhesion assay provides only a comparative ranking of bacterial surface adhesion and does not directly model adhesion to vaginal epithelial cells. Validation of the observed adhesion phenotypes in cervicovaginal epithelial-cell co-culture systems (e.g., VK2/E6E7) will therefore be required and is planned as part of the strain’s downstream characterization (see Section 4).

### 3.4. Species-Level Identification of Strain 127-4 by Whole-Genome Shotgun Sequencing

Genomic DNA was extracted from a purified colony of strain 127-4 and subjected to whole-genome shotgun sequencing. Quality-filtered reads were taxonomically classified using Kraken2 (v2.1.3) [22] against the standard bacterial reference database. Of the classified reads, 97.81% were assigned to *Lactiplantibacillus plantarum*, with the remaining 2.19% distributed among closely related members of the *Lactiplantibacillus* clade, consistent with a monoclonal *L. plantarum* culture and supporting preliminary species-level identification of strain 127-4 as *L. plantarum*. For consistency throughout the manuscript, the strain is hereafter referred to as *L. plantarum* 127-4. Raw sequencing data have been deposited in the NCBI Sequence Read Archive (SRA) under BioProject accession number PRJNA1470428.

### 3.5. L. plantarum 127-4 Is the Only Candidate with a Detectable Biofilm-Associated Phenotype

To distinguish strains capable of mucosal persistence from transient adherers, the four shortlisted candidates were evaluated in parallel for biofilm-associated phenotype using two complementary readouts: (i) crystal violet retention in 96-well polystyrene microplates measured spectrophotometrically at OD490, and (ii) qualitative imaging of glass-surface-associated structures by laser-capture microdissection (LCM) microscopy.

**Figure 4:**
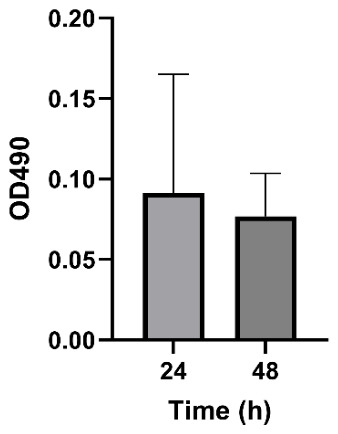
Biofilm formation of *L. plantarum* 127-4 after 24 and 48 h of anaerobic incubation, assessed by crystal violet retention. Bars represent mean OD490 ± SD of three independent experiments (*n* = 3). A detectable biofilm-associated signal was observed at both time points.

Of the four candidates, only strain 127-4 produced crystal violet retention above the blank-corrected baseline (Figure 4). The mean OD490 values for strain 127-4 were 0.09 ± 0.07 at 24 h and 0.08 ± 0.03 at 48 h, whereas the remaining three candidates yielded blank-corrected values at or below zero at both time points. The absolute biomass-associated signal of strain 127-4 falls at the lower end of published biofilm-formation reports for vaginal lactobacilli [8,11], and we therefore interpret it as evidence of early surface attachment with limited maturation rather than as the hyperbiofilm phenotype described for some allochthonous *L. plantarum* strains. Within the present screening framework, the signal observed for strain 127-4 was reproducible across three independent biological replicates and remained stable between 24 h and 48 h, indicating sustained rather than transient surface attachment under the applied experimental conditions.

Qualitative microscopic imaging of *L. plantarum* 127-4 on glass slides was consistent with the spectrophotometric findings: at 24 h, scattered surface-adherent cells and small aggregates were visible, whereas by 48 h these structures had progressed to larger cell clusters with faintly stained matrix-like material (Supplementary Figure S2). These observations document early surface attachment and aggregation under the applied experimental conditions but do not constitute evidence of mature biofilm architecture, which would require confocal laser scanning microscopy combined with exopolysaccharide-targeted staining (e.g., concanavalin A) and live/dead architectural analysis (SYTO9/propidium iodide). Such analyses are planned as part of the strain’s structural characterization in a dedicated follow-up study.

### 3.6. Integrated Functional Ranking Identifies L. plantarum 127-4 as the Priority Probiotic Candidate

To make the selection logic of the tiered screening workflow fully transparent, we generated a composite functional profile across all four shortlisted candidates (Supplementary Table S2). The analysis incorporated four parameters relevant to vaginal probiotic application: (i) breadth of antimicrobial activity (number of indicators inhibited at ≥ 5 mm halo diameter); (ii) maximum inhibition halo against any single indicator; (iii) SPA value as a proxy for surface adhesion; (iv) biofilm-associated OD490 at 48 h. The four candidates occupied distinct positions within this functional landscape:

- 127-3—broad antimicrobial spectrum (6/7 indicators, including the only anti-*S. agalactiae* activity), but low adhesion (SPA 1.86) and no detectable biofilm-associated signal;
- 107-2—strongest anti-*S. aureus* antimicrobial activity, but lowest adhesion (SPA 1.81) and no detectable biofilm-associated signal;
- 107-4—highest adhesion (SPA 3.39), but the narrowest antimicrobial spectrum (3/7 indicators) and no detectable biofilm-associated signal;
- 127-4—the only candidate combining broad antimicrobial activity (6/7 indicators), moderate adhesion (SPA 2.95), and a detectable biofilm-associated phenotype.

*L. plantarum* 127-4 was therefore the only isolate satisfying all three sequential functional criteria of the screening workflow and was selected as the priority candidate for downstream molecular and translational characterization. We note that strains 127-3 and 107-4 remain of interest as second-tier candidates with complementary protective profiles—127-3 because of its unique anti-*S. agalactiae* activity, which may be relevant to vertical transmission risk in obstetric settings, and 107-4 because of its comparatively strong adhesion phenotype. These isolates may therefore warrant future evaluation as components of a multi-strain consortium formulation alongside *L. plantarum* 127-4.

## 4. Discussion

### 4.1. Principal Findings and Contribution to the Field

In the present study, we applied a tiered functional screening workflow to a collection of 235 autochthonous vaginal lactic acid bacterial isolates recovered from a cohort of 400 Kazakhstani women and identified *Lactiplantibacillus plantarum* strain 127-4 as a probiotic candidate combining three functionally relevant traits: broad antimicrobial activity against urogenital pathogens, moderate surface adhesion, and a detectable biofilm-associated phenotype. To our knowledge, this study represents the first systematic functional characterization of cervicovaginal *Lactobacillus* and *Lactiplantibacillus* isolates from a Central Asian population, an underrepresented region in current cervicovaginal microbiome research despite a regional cervical cancer incidence approximately twice the European average [3]. Beyond identification of a single candidate strain, the study establishes a transferable workflow for functional annotation of autochthonous vaginal *Lactobacillus* collections, providing a framework for future population-specific probiotic strategies targeting cervicovaginal health [1,2].

### 4.2. Strain-Level Functional Heterogeneity Within the Autochthonous Cohort

The four shortlisted candidates exhibited markedly distinct phenotypic profiles despite their shared origin from a single geographically defined cohort. Strain 127-3 displayed the broadest antimicrobial spectrum and was the only candidate active against *Streptococcus agalactiae*; strain 107-2 demonstrated the strongest anti-*S. aureus* antimicrobial activity; strain 107-4 exhibited the highest adhesion phenotype; and only strain 127-4 combined antimicrobial, adhesive, and biofilm-associated traits within a single isolate. This phenotypic divergence among isolates that would otherwise be classified identically at the species level reinforces a now well-established principle in probiotic science: probiotic functionality is primarily a strain-level rather than species-level property [9,20].

The implications for vaginal probiotic development are twofold. First, species-level identification—although a regulatory minimum requirement—is insufficient to predict clinical performance. Second, single-criterion screening workflows, which continue to dominate much of the published literature, may overlook strains whose value lies in complementary trait combinations rather than in a single dominant phenotype. Antimicrobial activity, epithelial adhesion, and biofilm-associated persistence are widely regarded as core functional determinants of vaginal probiotic performance and mucosal colonization capacity [2,8,9,10]. Because these traits frequently vary independently even among isolates belonging to the same species, multi-trait screening frameworks have increasingly been advocated for rational vaginal probiotic prioritization.

The composite framework applied in the present study was therefore designed as an exploratory candidate-prioritization strategy integrating complementary probiotic-relevant phenotypes rather than as a validated clinical scoring system. Importantly, the purpose of the composite framework was not to generate a universal ranking score, but to provide a transparent and reproducible method for integrating multiple functional traits during candidate selection. Because vaginal probiotic performance depends on the combined contribution of pathogen inhibition, host-surface interaction, and persistence-associated characteristics, prioritization based on a single phenotype may overlook strains with balanced probiotic potential. The present framework was therefore designed to support comparative evaluation of shortlisted candidates within a defined isolated collection rather than to serve as a standalone predictive model of clinical efficacy. To our knowledge, no universally accepted ranking system currently exists for vaginal probiotic candidate prioritization, highlighting the need for transparent multi-parameter approaches that facilitate rational strain selection. The tiered screening workflow applied here may also have broader applicability beyond the cervicovaginal niche in future mucosal probiotic discovery programs.

### 4.3. Antagonistic Activity: Spectrum and Mechanistic Considerations

The marked stratification of inhibitory activity by indicator type—with Gram-negative pathogens demonstrating broader susceptibility than Gram-positive bacteria or yeasts—is consistent with previous reports for vaginal lactobacilli [1,11] and likely reflects the dominant contribution of organic acid-mediated inhibition under the deferred antagonism format used here, which does not buffer the agar surface to physiological pH. We acknowledge that this format does not resolve the relative contributions of lactic acid, hydrogen peroxide, and bacteriocin-like inhibitory substances to the observed antimicrobial activity. However, the observation that some candidates (e.g., strain 127-3) demonstrated activity against *S. agalactiae*—a pathogen relatively resistant to lactic acid-mediated killing within the pH range achievable in MRS-based assays [23]—suggests that non-acid antimicrobial metabolites contribute to the antimicrobial spectrum of at least a subset of the isolates. Differential characterization of these contributions using pH-controlled and neutralized cell-free supernatant assays will be essential for resolving the molecular basis of inhibition and guiding rational formulation strategies and is planned as part of the strain’s mechanistic follow-up.

The observation that strain 127-4 produced large inhibition halos against enterobacterial indicator strains (>33 mm against both *E. coli* and *E. faecalis*), but comparatively modest activity against yeast indicators, is consistent with antimicrobial profiles previously reported for vaginal- and gut-derived *L. plantarum* strains [24,25]. These findings support the interpretation that strain 127-4 belongs to the broad-spectrum subset of *L. plantarum* isolates with potential translational relevance for urogenital dysbiosis.

### 4.4. Adhesion and Biofilm-Associated Phenotype: Interpretation Within the Screening Framework

Among the four shortlisted candidates, *L. plantarum* 127-4 was the only isolate displaying both moderate adhesion (SPA 2.95) and a reproducible biofilm-associated signal under the applied static microtiter-plate conditions. The combination of these two traits is functionally relevant because mucosal persistence within the vaginal niche is thought to depend on coordinated surface attachment and matrix-associated retention rather than on either trait alone [10,26].

The absolute biofilm-associated signal observed for strain 127-4 (OD490 = 0.09 ± 0.07 at 24 h; 0.08 ± 0.03 at 48 h) falls at the lower end of published biofilm-formation reports for vaginal lactobacilli [8,11]. We therefore interpret this signal as evidence of early surface attachment with limited matrix maturation under the applied static conditions rather than as the hyperbiofilm phenotype described for some allochthonous *L. plantarum* strains. Several factors may have contributed to this relatively modest signal. First, strain 127-4 was assayed in standard MRS broth on polystyrene surfaces, neither of which adequately reflects the physicochemical conditions of the vaginal niche, including mucin, glycogen-derived substrates, lower pH, and microaerophilic-to-anaerobic gradients; *L. plantarum* biofilm formation is known to be highly substrate- and surface-dependent [26]. Second, static 24–48 h assays may underestimate the biofilm-associated capacity of strains adapted to dynamic mucosal environments. Third, the absence of reference biofilm-forming and non-biofilm-forming control strains—addressed below in Section 4.6—precluded normalization of the observed signal against a defined biological reference scale.

These considerations argue not for dismissing the biofilm-associated phenotype of strain 127-4, but rather for re-evaluating it under conditions that better recapitulate the vaginal mucosal environment. In particular, future analyses should include: (i) inclusion of *L. plantarum* WCFS1 or comparable reference strains alongside known non-biofilm-forming controls; (ii) substitution of polystyrene surfaces with mucin-coated substrates or vaginal epithelial-cell monolayers; and (iii) confocal laser scanning microscopy combined with exopolysaccharide-targeted staining (e.g., concanavalin A) and live/dead architectural analysis. These approaches are planned as part of the strain’s structural characterization in a dedicated follow-up study.

### 4.5. Translational Positioning of L. plantarum 127-4

*Lactiplantibacillus plantarum* is among the most extensively characterized probiotic species and is known for its metabolic versatility, broad-spectrum antimicrobial activity, and well-established safety profile across multiple commercial probiotic formulations [24,25]. However, the species generally represents only a minor component of the autochthonous vaginal microbiota in most populations, and many vaginal-associated *L. plantarum* strains characterized to date have originated from food-fermentation collections or gastrointestinal sources. Recent comparative studies have demonstrated that vaginal-niche *L. plantarum* isolates may exhibit distinct adhesion, antimicrobial, and immunomodulatory phenotypes relative to gut- and food-derived counterparts [24], reinforcing the rationale for identifying autochthonous vaginal *L. plantarum* candidates rather than repurposing strains from non-vaginal niches.

Within this context, *L. plantarum* 127-4 occupies a potentially relevant translational position as an autochthonous vaginal isolate derived from an underrepresented population and displaying a balanced functional profile. The strain may therefore be suitable for future development either as a single-strain probiotic candidate or, potentially more importantly, as part of a defined multi-strain consortium alongside second-tier candidates 127-3 and 107-4. In particular, strain 127-3 demonstrated unique anti-*S. agalactiae* activity, which may be relevant to vertical transmission risk in obstetric settings, whereas strain 107-4 exhibited the strongest adhesion-associated phenotype among the shortlisted candidates. Defined microbial consortia are increasingly recognized as a potentially more robust strategy than single-strain formulations for restoration of complex mucosal microbial communities [9]. The present isolate collection therefore provides a candidate foundation for future consortium-based probiotic development pending downstream functional validation.

### 4.6. Limitations

Several limitations should be considered when interpreting these findings and defining priorities for follow-up work. All assays were performed in vitro, and the deferred antagonism format does not differentiate the relative contributions of organic acid production, H_2_O_2_, and bacteriocin-like inhibitory substances to the observed antimicrobial activity; pH-controlled neutralized supernatant assays are therefore planned. Crystal violet retention was measured at OD_490_ for internal consistency, limiting cross-study comparability with assays performed at 570/595 nm. In addition, reference positive and negative biofilm-control strains were not included, limiting discrimination between low-level early attachment and assay-background signal; their inclusion in future studies will be essential for biological normalization and interpretation of weak OD_490_ values. Microscopic imaging supports qualitative interpretation only, and confocal laser scanning microscopy with matrix-component staining will be required for definitive architectural characterization.

The Brilis erythrocyte assay provides comparative ranking rather than a direct model of vaginal epithelial colonization and therefore represents a semi-quantitative surrogate assay dependent on erythrocyte receptor specificity rather than epithelial-cell interaction. Consequently, the relatively low-to-moderate SPA values observed in the present study should primarily be interpreted comparatively within the same experimental framework. Previous methodological studies based on erythrocyte adhesion models have noted that adhesion indices may differ substantially from those obtained using epithelial-cell systems because of differences in receptor availability and surface architecture [16]. A reference high-adhesion control strain was not included in the present exploratory screening study, and its inclusion will be important for future biological normalization. Validation in cervicovaginal epithelial-cell co-culture systems (VK2/E6E7, Ect1/E6E7) and mucin-coated flow systems is planned.

Species-level assignment currently rests on Kraken2 k-mer classification (97.81% assigned to *L. plantarum*); full-length 16S rRNA sequencing, ANI analysis against WCFS1, complete genome assembly, and CARD/RGI and VFDB screening are in progress [20], and species identification should therefore be considered preliminary until these analyses are completed. As a screening workflow, the present study reports descriptive statistics only; HPV status, CST membership, and clinical phenotype associations will be analyzed in a subsequent population-level investigation.

### 4.7. Future Directions and Translational Outlook

*L. plantarum* 127-4 will be advanced through complete genomic characterization, including ANI confirmation and antimicrobial resistance/virulence-factor screening, alongside mucosal-relevant functional validation in cervicovaginal epithelial-cell systems and assessment of IL-1β/IL-17 immunomodulatory activity [6]. Additional translational evaluation in cervicovaginal explant models is planned. The workflow provides a transferable framework for population-specific probiotic candidate selection in HPV-related cervicovaginal disease [4,27].

## 5. Conclusions

Tiered functional screening of 235 autochthonous vaginal lactic acid bacterial isolates recovered from a cohort of Kazakhstani women identified *L. plantarum* 127-4 as the only candidate combining broad antimicrobial activity, moderate surface adhesion, and a detectable biofilm-associated phenotype within a single isolate. The study establishes the first functionally characterized cervicovaginal *Lactobacillus/Lactiplantibacillus* collection from a Central Asian population and provides a transferable workflow for population-specific probiotic candidate identification. *L. plantarum* 127-4, alongside second-tier candidates 127-3 and 107-4, provides a foundation for downstream characterization addressing genomic safety, mucosal-relevant functional validation, and future translational development in accordance with current FAO/WHO and ISAPP recommendations for probiotic strain characterization.

## Data Availability

The whole-genome shotgun sequencing data generated in this study have been deposited in the NCBI Sequence Read Archive under accession number PRJNA1470428. Additional data supporting the findings of this study are available from the corresponding authors upon reasonable request.

## Supplementary Materials

The following supporting information can be downloaded at: https://www.mdpi.com/article/10.3390/microorganisms14071526/s1. Figure S1: Morphological and microscopic characteristics of vaginal *Lactobacillus* isolates 107-2 and 127-4. (A,B) Colony morphology of isolates grown on MRS agar. Colonies were small, non-pigmented, convex, with smooth surfaces and regular margins. (C,D) Representative Gram-stained images of isolates 107-2 (C) and 127-4 (D), showing Gram-positive rod-shaped cells (light microscopy, ×1000); Figure S2: Surface-associated structures of *L. plantarum* 127-4 after 24 and 48 h of incubation, visualized by laser-capture microdissection (LCM). (A) Representative image at 24 h showing adherent bacterial cells and small aggregates. (B) Representative image at 48 h showing larger aggregates with faint matrix-like material. Scale bars are indicated in the images; Table S1: Antimicrobial activity profiles of the 37 retained vaginal Lactobacillus isolates against seven urogenital indicator microorganisms; Table S2: Composite functional profile of shortlisted vaginal isolates.

## Abbreviations

ANI	Average Nucleotide Identity
CST	Community State Type
HPV	Human Papillomavirus
ISAPP	International Scientific Association for Probiotics and Prebiotics
LCM	Laser-Capture Microdissection
MRS	de Man, Rogosa and Sharpe medium
OD	Optical Density
SPA	Specific Adhesion Index
SRA	Sequence Read Archive
UMC	University Medical Center
