# Supplementary material for: Tiered Functional Screening Identifies an Autochthonous Vaginal Lactiplantibacillus plantarum Strain with Probiotic Potential
Source: Microorganisms. 2026 Jul 13;14(7):1526. doi: 10.3390/microorganisms14071526 (PMC13414338; doi:10.3390/microorganisms14071526)
Supplement: Supplementary file 1 [file microorganisms-14-01526-s001.zip › microorganisms-4334304-supplementary.pdf]

Supplementary Materials:

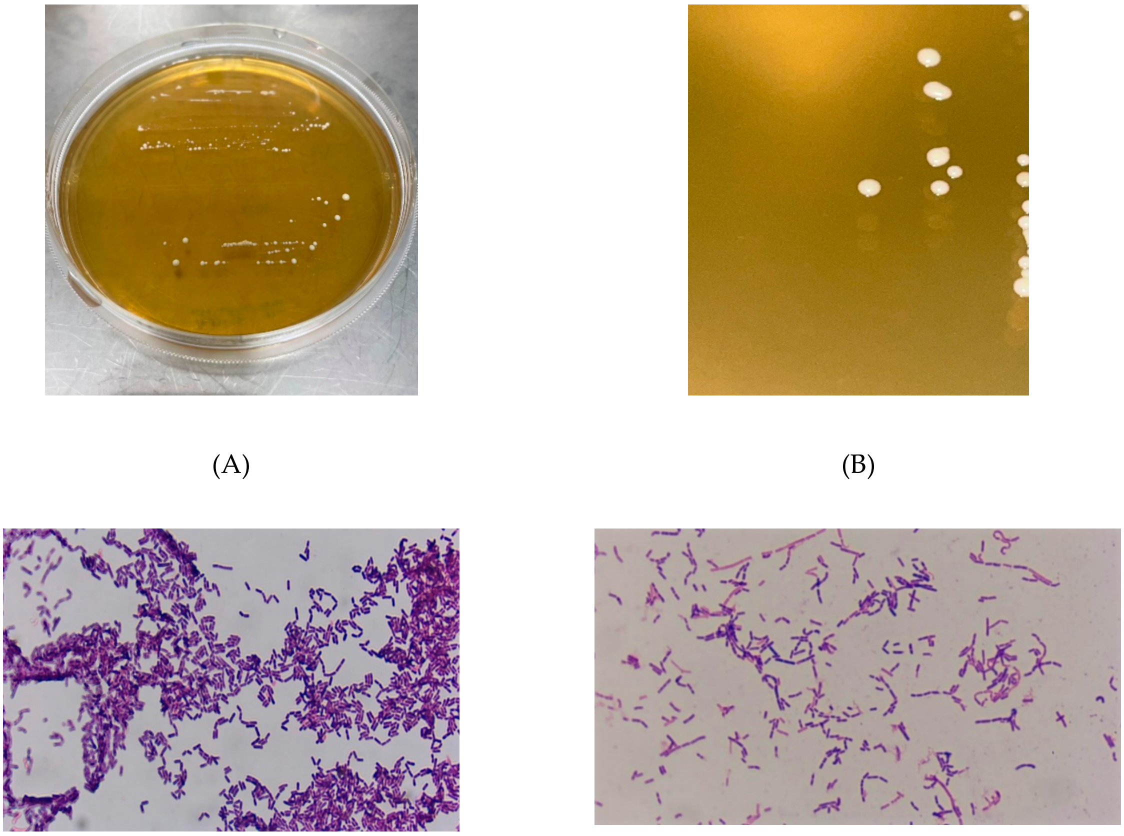

(A) (B)

(C) (D)

Figure S1. Morphological and microscopic characteristics of vaginal *Lactobacillus* isolates 107-2 and 127-4. (A,B) Colony morphology of isolates grown on MRS agar. Colonies were small, non-pigmented, convex, with smooth surfaces and regular margins. (C,D) Representative Gram-stained images of isolates 107-2 (C) and 127-4 (D), showing Gram-positive rod-shaped cells (light microscopy, ×1000).

Table S1. Antimicrobial activity profiles of the 37 retained vaginal Lactobacillus isolates against seven urogenital indicator microorganisms.

**Sample ID**	**Isolate ID**	***E. coli* ATCC 25922 Mean ± SD (mm)**	***E. faecalis* ATCC 19433 (mm)**	***C. albicans* ATCC 10231 (mm)**	***S. aureus* ATCC 29213 (mm)**	***C. glabrata* ATCC 15126 (mm)**	***P. mirabilis* ATCC 25933 (mm)**	***S. agalactiae* ATCC 13813 (mm)**
		**Mean ± SD (mm)**						
	16-3	11.00 ± 1.00	14.00 ± 0.50	3.00 ± 1.00	7.00 ± 0.50	0	0	0
	16-5	12.00 ± 0.50	11.50 ± 0.50	3.00 ± 2.00	3.50 ± 0.50	0	0	0
	16-6	15.50 ± 0.50	11.50 ± 1.50	2.00 ± 0.50	8.50 ± 2.00	0	0	0

	21-8	12.00 ± 0.50	9.00 ± 1.00	0	19.50 ± 0.50	0	0	0
	21-10	8.50 ± 1.00	11.00 ± 1.00	4.00 ± 2.00	19.00 ± 3.00	0	0	0
	21-11	9.50 ± 0.50	4.50 ± 0.50	4.00 ± 0.50	19.00 ± 0.50	0	0	0
**107**	107-2	19.00 ± 1.00	26.00 ± 0.50	21.00 ± 3.00	30.50 ± 0.50	6.00 ± 2.00	71.00 ± 0.50	0
	107-4	0	0	8.00 ± 0.50	0	16.00 ± 0.50	64.00 ± 3.00	0
**127**	127-1	27.67 ± 0.58	30.50 ± 1.50	16.50 ± 0.50	0	8.00 ± 2.00	52.00 ± 3.00	0
	127-3	30.00 ± 1.00	35.83 ± 2.02	16.50 ± 0.50	0	9.00 ± 0.50	43.00 ± 3.00	17.50 ± 0.50
	127-4	33.67 ± 0.58	33.50 ± 0.50	14.50 ± 3.00	21.00 ± 3.00	5.50 ± 2.00	64.00 ± 0.50	0
**297**	297-1	18.50 ± 1.50	13.00 ± 3.00	14.00 ± 0.50	29.50 ± 3.00	4.00 ± 1.00	53.50 ± 3.00	0
	297-4	16.00 ± 1.00	4.00 ± 0.50	13.50 ± 3.00	9.50 ± 0.50	9.00 ± 0.50	67.00 ± 0.50	0
**301**	301-1	20.67 ± 0.58	15.00 ± 0.50	0	14.50 ± 3.00	0	25.00 ± 3.00	0
**307**	307-8	9.67 ± 1.53	15.50 ± 3.00	3.50 ± 0.50	10.00 ± 0.50	0	11.50 ± 3.00	0
**309**	309-1	11.67 ± 1.04	12.50 ± 3.00	5.00 ± 2.00	18.00 ± 3.00	0	29.50 ± 0.50	0
	309-2	13.67 ± 0.58	12.00 ± 1.73	5.50 ± 0.50	13.50 ± 0.50	0	28.00 ± 3.00	15.00 ± 0.50
	309-3	16.67 ± 1.26	14.00 ± 0.50	5.00 ± 1.25	14.00 ± 0.50	0	40.50 ± 0.50	18.00 ± 1.50
	309-4	13.67 ± 1.53	12.50 ± 3.00	5.00 ± 0.50	15.00 ± 3.00	0	15.00 ± 3.00	20.50 ± 0.50
**316**	316-1	28.67 ± 1.04	36.50 ± 3.00	13.00 ± 3.00	30.00 ± 0.50	0	42.00 ± 0.50	0
	316-3	29.00 ± 1.00	36.50 ± 2.00	11.50 ± 0.50	26.00 ± 0.50	0	35.00 ± 3.00	0
**317**	317-1	13.00 ± 1.00	14.00 ± 1.00	4.00 ± 0.50	9.00 ± 0.50	0	33.50 ± 3.00	20.00 ± 3.00
	317-2	9.50 ± 1.00	12.00 ± 1.00	4.00 ± 2.00	15.00 ± 3.00	0	17.00 ± 0.50	0
**321**	321-1	13.67 ± 0.58	12.00 ± 3.00	0	10.00 ± 0.50	0	10.00 ± 3.00	0
	332-3	8.50 ± 0.50	10.50 ± 0.50	5.00 ± 3.00	9.00 ± 3.00	0	17.50 ± 0.50	0
**336**	336-1	10.00 ± 0.50	14.00 ± 3.00	5.00 ± 3.00	14.00 ± 1.00	0	28.00 ± 3.00	0

	337-6	11.50 ± 0.50	12.00 ± 3.00	0	24.00 ± 0.50	0	19.50 ± 3.00	0
**346**	346-1	21.67 ± 0.58	13.00 ± 0.50	6.50 ± 0.50	14.00 ± 3.00	0	0	0
	346-3	16.00 ± 0.50	10.50 ± 0.50	5.50 ± 2.00	11.50 ± 3.00	0	0	0
**354**	354-1	12.67 ± 0.58	9.50 ± 3.00	0	13.00 ± 0.50	0	0	0
	375-3	25.17 ± 0.76	26.50 ± 3.00	3.50 ± 2.00	13.50 ± 3.00	0	0	0
**377**	377-1	26.00 ± 1.00	10.50 ± 0.50	11.50 ± 3.00	19.00 ± 3.00	0	40.00 ± 3.00	0
	377-2	26.00 ± 1.00	30.00 ± 3.00	15.50 ± 0.50	22.50 ± 0.50	7.00 ± 0.50	0	0
**384**	384-1	11.00 ± 1.00	10.67 ± 2.75	3.00 ± 0.50	13.50 ± 2.50	0	39.50 ± 3.00	0
	384-2	11.67 ± 0.58	10.00 ± 3.00	0	6.50 ± 0.50	0	32.50 ± 0.50	0
**392**	392-1	34.00 ± 1.00	34.00 ± 3.00	13.00 ± 1.00	31.00 ± 3.00	0	51.00 ± 0.50	0
	392-2	31.67 ± 1.04	30.50 ± 0.50	11.00 ± 0.50	27.50 ± 0.50	7.00 ± 3.00	47.50 ± 3.00	17.50 ± 0.50

Values are presented as mean inhibition-zone diameters ± SD (mm) obtained from three independent experiments. A value of 0 indicates absence of detectable inhibitory activity.

Table S2. Composite functional profile of shortlisted vaginal isolates.

Isolate ID	Antimicrobial breadth (≥5 mm)	Max inhibition halo (mm)	SPA (mean ± SD)	Biofilm OD490 (48 h)
127-3	6/7	43.0	1.86 ± 0.46	0
127-4	6/7	64.0	2.95 ± 0.53	0.08
107-2	6/7	71.0	1.81 ± 0.24	0
107-4	3/7	64.0	3.39 ± 0.62	0

Antimicrobial breadth indicates the number of inhibited indicator strains (≥5 mm). Maximum inhibition halo corresponds to the largest observed mean halo diameter. SPA values are presented as mean ± SD (n = 3). Biofilm formation is expressed as OD490 at 48 h; values ≤0 after blank correction are shown as zero.

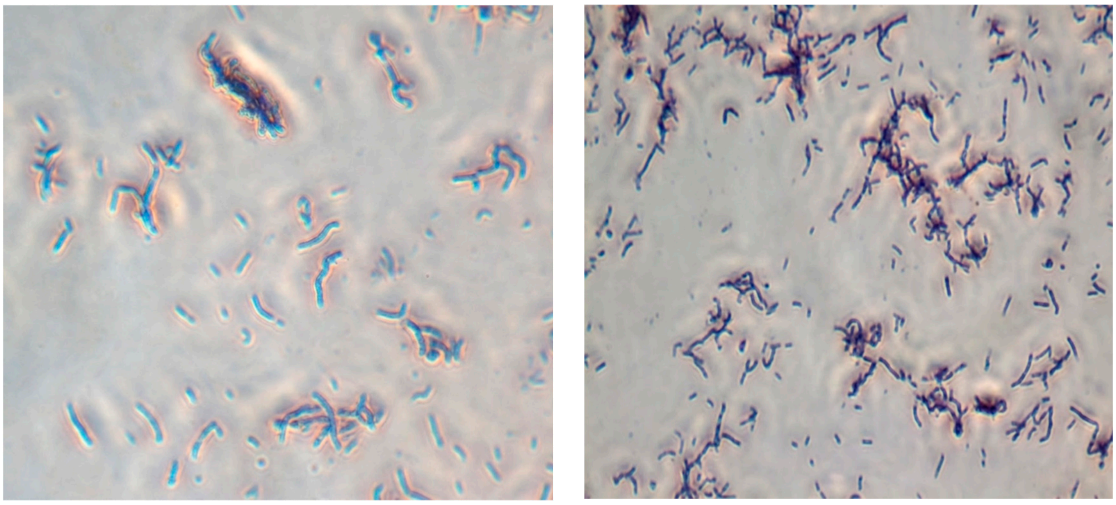

(A) (B)

Figure S2. Surface-associated structures of *L. plantarum* 127-4 after 24 and 48 h of incubation, visualized by laser-capture microdissection (LCM). (A) Representative image at 24 h showing adherent bacterial cells and small aggregates. (B) Representative image at 48 h showing larger aggregates with faint matrix-like material. Scale bars are indicated in the images.
